# The Role of microRNAs in Multidrug Resistance of Glioblastoma

**DOI:** 10.3390/cancers14133217

**Published:** 2022-06-30

**Authors:** Parvaneh Mahinfar, Behnaz Mansoori, Davoud Rostamzadeh, Behzad Baradaran, William C. Cho, Behzad Mansoori

**Affiliations:** 1 Immunology Research Center, Tabriz University of Medical Sciences, Tabriz 5166/15731, Iran; parvaneh.mahinfar2020@gmail.com (P.M.); behnaz.mansoori@gmail.com (B.M.); baradaranb@tbzmed.ac.ir (B.B.); 2 Department of Molecular Genetics, Faculty of Biological Sciences, Tarbiat Modares University, Tehran 175-14115, Iran; 3 Department of Clinical Biochemistry, Yasuj University of Medical Sciences, Yasuj 7591994799, Iran; d.rostamzadeh@yahoo.com; 4 Medicinal Plants Research Center, Yasuj University of Medical Sciences, Yasuj 7591994799, Iran; 5 Department of Clinical Oncology, Queen Elizabeth Hospital, Kowloon, Hong Kong SAR, China; 6 The Wistar Institute, Molecular & Cellular Oncogenesis Program, Philadelphia, PA 19104, USA

**Keywords:** glioblastoma, multidrug resistance, microRNAs, drug transporters, metabolism, DNA repair, epithelial–mesenchymal transition, apoptosis

## Abstract

**Simple Summary:**

Glioblastoma (GBM) is one of the most malignant types of central nervous system tumor which accounts for more than 60% of all brain tumors in adults. Owing to poor prognosis and drug resistance of most GBM, it is urged to further develop the diagnosis and treatment strategies. The aim of this article is to highlight the roles of some functional microRNAs in the diagnosis and treatment of drug-resistant GBM. Besides, we suggest effective treatment strategies based on the expression profiles of these effective miRNAs to provide an alternative solution to deal with this cancer.

**Abstract:**

Glioblastoma (GBM) is an aggressive brain tumor that develops from neuroglial stem cells and represents a highly heterogeneous group of neoplasms. These tumors are predominantly correlated with a dismal prognosis and poor quality of life. In spite of major advances in developing novel and effective therapeutic strategies for patients with glioblastoma, multidrug resistance (MDR) is considered to be the major reason for treatment failure. Several mechanisms contribute to MDR in GBM, including upregulation of MDR transporters, alterations in the metabolism of drugs, dysregulation of apoptosis, defects in DNA repair, cancer stem cells, and epithelial–mesenchymal transition. MicroRNAs (miRNAs) are a large class of endogenous RNAs that participate in various cell events, including the mechanisms causing MDR in glioblastoma. In this review, we discuss the role of miRNAs in the regulation of the underlying mechanisms in MDR glioblastoma which will open up new avenues of inquiry for the treatment of glioblastoma.

## 1. Introduction

Glioblastoma is a malignant brain tumor which develops from neuroglial stem cells [1,2]. According to the fifth edition of the WHO classification of Central Nervous System (WHO CNS5) tumor in 2021, 14 distinct tumors have been identified. This alteration incorporates numerous molecular changes with clinicopathologic utility for the most accurate classification of CNS neoplasms. On the basis of a recent version of WHO CNS5, fundamental changes in molecular and practical approaches including histology and immunohistochemistry to CNS tumor taxonomy were added and introduced the role of molecular diagnostics in CNS tumor classification. The momentous changes in WHO CNS5 involve the classification of gliomas, differentiating gliomas that occur primarily in adults. In fact, in adults, diffuse gliomas have been categorized into three types including astrocytoma, IDH-mutant; oligodendroglioma, IDH-mutant, 1p/19q-co-deleted; and glioblastoma, IDH-wildtype. Additionally, in WHO CNS5, glioblastomas will comprise only IDH-wildtype tumors. In the new classification, all IDH-mutant diffuse astrocytic tumors are considered a single type (astrocytoma, IDH-mutant) and are graded as 2, 3, or 4 [3,4]. It is estimated that the 5-year survival rate for glioblastomas is less than 5% [3]. Although, there have been significant improvements in both the research and development for combating this type of cancer, the prognosis and long-term outlook for patients with glioblastoma remain poor [5]. Glioblastoma treatment failure is thought to be due to its anatomical location, and the presence of the blood–brain barrier which hinders the transport of chemotherapeutic agents. Commute inhibition of immune cells results in aggressive tumor cell behavior [6]. One of the main reasons why glioblastoma treatments have failed is the occurrence of multi-drug resistance (MDR) to common chemotherapy drugs. According to recent information that release through FDA-approved chemotropic drugs, there are four drugs for the treatment of glioblastoma: temozolomide (TMZ), lomustine, intravenous carmustine, and bevacizumab (BVZ). As mentioned above, only TMZ, tumor treatment fields, is approved for new diagnoses in high-grade gliomas (HGG). There is no standard of care (SOC) for these reversions. Moreover, only MDR to TMZ has been reported in glioblastoma malignancy [7,8]. According to recent studies, over 90% of cancer patients who die due to drug resistance are receiving traditional chemotherapeutics or novel targeted drugs [9]. In response to chemotherapy, a large number of tumor cells become resistant to the amount of drug administered [9,10]. It is widely acknowledged that drug resistance is a significant health issue that adversely affects cancer treatment effectiveness [11]. It has been found that a variety of molecular mechanisms are involved in the development of MDR, such as dysregulation of drug efflux proteins expression, modification of drug targets, disruption of cell cycle checkpoints, altered apoptosis, enhanced DNA damage repair in cancer stem cells, and mediators of posttranscriptional regulation, such as microRNAs (miRNAs) [12]. There are numerous biomarkers, such as biological markers, found in body fluids which assume an essential role in many aspects of oncology such as diagnosis. These biomarkers are signals of the cancer’s presence and consist of molecular alteration, proliferation, or process in the patient’s body. For instance, an alteration in the expression of several proteins may indicate the presence of cancer. The prognosis of GBM patients is poor in most cases. Therefore, utilizing some specific and sensitive biomarkers is a great approach to the diagnosis of GBM. According to the recent studies, there are some putative biomarkers of glioblastoma such as CD133, CD44, CD15, CD70, S100A4, ALDH1A3, NANOG, OCT-4, and SOX-2. Furthermore, all of these biomarkers consider MDR GBM because of fluctuations in expression, especially in glioblastoma stem cells (GSCs). GSCs make a great impact on chemotherapy resistance and cause tumor recurrence. These markers are associated with cascading pathways and interactions with some important and functional miRNAs such as *miR-20a* which are upregulated in MDR GBM. In addition, *CCL22*, *ADCY2*, *PDK1*, *ZFP36*, *CP*, *CD2*, *PLAUR*, *ACAP1*, *COL5A1*, *FAM83D*, *PBK*, *FANCA*, *ANXA7*, and *TACC3* were identified as genetic biomarkers that were all associated with pathways in GBM progression and MDR. Investigation of the expression these hallmarks is one of the appropriate methods to detect cancer cells in the early phase [13,14]. Among these, in general, miRNAs are small (usually 19–24 nucleotides) non-coding RNAs that play an important role in various critical cellular processes by targeting and modulating the expression of key genes involved [15]. MiRNAs play a key regulatory role in MDR through modulating various drug resistant mechanisms that are more significant in current treatment approaches. In particular, miRNAs that are deregulated play a momentous role in glioblastoma, in which they participate in multiple cellular processes, including proliferation, apoptosis, autophagy, invasion, metastasis, and angiogenesis [16,17]. This review aimed to highlight the importance of miRNAs in the regulation of MDR-related mechanisms.

## 2. MDR in GBM

A major cause of chemotherapy treatments failure is MDR, the mechanism by which cancers become resistant to chemotherapy drugs. A major cause of many chemotherapy treatments failing is MDR, the mechanism by which cancers become resistant to chemotherapy drugs. Different kinds of chemotherapy agents are used to treat cancer—either alone or in combination with other agents. These agents are various in their chemical composition. The mainly chemotropic agents include antimetabolites (5-fluorouracil (5-FU)), alkylating agents (temozolomide, cisplatin), topoisomerase inhibitors (doxorubicin), and mitotic spindle inhibitors (paclitaxel) [9]. The development of MDR in GBM has been linked to numerous molecular mechanisms including upregulation of MDR transporters, changes in the metabolism of drugs, dysregulation of apoptosis, defects in DNA repair, cancer stem cells, and epithelial-mesenchymal transition (EMT).

### 2.1. Upregulation of MDR Transporters

Increasing the efflux of drugs in cancer cells, especially in glioblastoma cells is one of the most significant and well-known mechanisms for developing MDR [18,19]. ATP binding cassette (ABC) transporter family members are considered to be essential transmembrane proteins which play a significant role in MDR with respect to pumping chemotherapeutic agents from tumor cells into the extracellular space as a result of ATP cleavage [20,21]. Therefore, these transporters diminish the cytotoxicity of anticancer agents by reducing the intracellular concentration of drugs. Among 48 members of ABC transporter members P-glycoprotein (P-gp/ABCB1) a transmembrane glycoprotein, and multidrug resistance-associated protein-1 (MRP1/ABCC1) are two extensively investigated members in GBM MDR [22]. Various studies have shown that these proteins are involved in the development of resistance against chemotherapeutic agents in this type of cancer. Moreover, co-localization of both P-gp and ABCG2 proteins is observed in glioblastoma cells, which is correlated to their joint functioning as drug transporters [19]. Consequently, blocking or inactivating ABC transporters increases the concentration of anti-neoplastic drugs in a cell [23].

### 2.2. Changes in the Metabolism of Drugs

The alteration of antitumor drug metabolism is another important mechanism used by glioblastoma cancer cells to reduce the cytotoxic effects of chemotherapy. Cytochrome P450 (CYP) enzymes which are expressed at higher levels in tumors of the digestive tract, liver, kidney, and brain, play a significant role in the MDR against vincristine, taxanes, etoposide, vinblastine, ifosfamide, doxorubicin, irinotecan, and cyclophosphamide [24]. Upregulating the expression of drug-metabolizing CYP (17A1) which catalyzes the metabolism of cholesterol to neurosteroids in GBM, causes MDR during treatment [25,26]. Many factors that affect CYP activities, including genetic polymorphisms, diseases, medications, certain foods, physiological conditions, and smoking, can alter pharmacokinetic profiles which are affecting chemotherapeutic efficacy in glioblastoma tumor cells [27].

### 2.3. Dysregulation of Apoptosis

As a consequence of chemotherapeutic-induced DNA damage, cancer cells can undergo two biological events, including cell cycle arrest and DNA repair, or apoptosis and cell death. During this process, TP53 plays a fundamental role, particularly during GBM MDR [28,29]. TP53 mutations in GBM mainly point to mutations that promote the development of MDR [30]. Normally, the most effective way of killing cancer cells is by inducing apoptosis with chemotherapeutic agents such as DNA cross-linking agents, antimetabolites, topoisomerase I/II inhibitors, and intercalating agents, and tyrosine kinase inhibitors (TKIs).

The disruption of apoptotic pathways, both intrinsic and extrinsic, has been implicated in the development of glioma MDR [31]. Tumor cells can evade apoptosis via downregulating pro-apoptotic proteins and upregulating anti-apoptotic proteins. The escape of apoptosis has been widely observed in MDR against a variety of chemotherapeutics, including paclitaxel, doxorubicin, mitoxantrone, etoposide, cisplatin, and camptothecin [32,33,34].

### 2.4. Defects in DNA Repair

As a consequence of chemotherapy-induced DNA damage, cancer cells have increased DNA repair capability which is implicated in MDR development. At the beginning of gliomagenesis, the DNA damage repair (DDR) system is fundamentally activated via oncogene-evoked replication and oxidative stress. By reason of, DNA repair machinery diminishes the efficacy of genotoxic treatments, understanding and characterizing the DDR is essential to developing new therapeutic strategies in GBM [35]. A recent study reported that combining chemotherapeutics with DNA repair inhibitors helps improve treatment efficacy by decreasing the likelihood of chemo-resistant cancer arising [36].

### 2.5. Cancer Stem Cells

Cancer stem cells (CSCs) are subpopulations of tumor cells that are characterized by the ability to differentiate and self-renew, aberrant proliferation, long lifespan, active DNA repair capacity, and resistance to apoptosis [37]. As a result of their specific intrinsic mechanisms, CSCs do not disappear with chemotherapy because they overexpress drug transporters which prevent them from undergoing anti-cancer agent-induced apoptosis [38]. GSC are multipotent cells that share many of the characteristics of CSCs, such as their capacity for self-renewal. Moreover, they are evidence of a critical role in tumor maintenance, recurrence, and the development of glioblastoma MDR [39,40]. Additionally, CSC-like populations of glioblastoma cells are highly resistant to glioblastoma therapeutic agents [39].

### 2.6. Epithelial to Mesenchymal Transition

**Figure 1:**
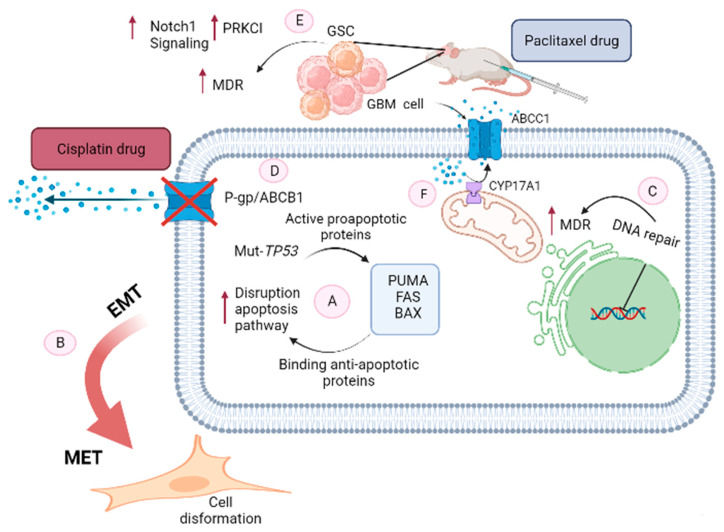
Mechanisms of MDR development in glioblastoma. A. Mutant*TP53*; the two accepted pathways leading to *TP53*-mediated apoptosis are exogenous and endogenous. In endogenous apoptosis, *TP53* activates pro-apoptotic proteins PUMA, FAS, and BAX. In response to activation of these proteins, *TP53* binds to apoptotic proteins from the *Bcl*-2 family and finally induces cytochrome c/Apaf-1-dependent endogenous apoptosis. In contrast, mutations in the *TP53* gene in cancers such as glioblastoma can cause PUMA, FAS, and BAX proteins to bind to anti-apoptotic proteins and result in the development of MDR. B. EMT; EMT is a subprocess that occurs during the progression of cancer which alters the morphology of cancer cells into highly motile and elongated mesenchymal-like cells which increases the capacity of tumor cells to resist chemotherapy. C. DNA repair; many chemotherapeutic drugs damage DNA in a manner that causes cell cycle arrest and cell death. Therefore, DNA repair involves an intricate network of repairing the cell, and that process leads to the development of MDR. D. P-gp /ABCB1 as a transmembrane protein causing lowered drug accumulation inside cells and consequently diminished drug efficacy. E. In glioblastoma stem-like cells (GSC), *Notch1* and *PRKCI* are overexpressed and are associated with MDR. F. CYP (17A1) as a drug metabolizer overexpressed in glioblastoma and efflux the chemotropic drug through ABCC1 transporter.

Another possible mechanism for the development of MDR in cancer cells is the converting EMT is a reversible biological process to mesenchymal–epithelial transition (MET) characteristics [41]. In this complex process, some significant morphologic alterations occur in epithelial cells which are transformed into elongated fibroblastic mesenchymal cells phenotype, that are significantly more invasive and motile, as well as developing an MDR [42]. Such diversity in mesenchymal features may be brought about by various microenvironmental factors, as well as intrinsic genetic alterations in glioma tissues. Simultaneous with genetic and especially epigenetic alterations that cancer cells endure making them sensitive to EMT-inducing signals. Mesenchyme-like cancer cells are commonly observed at the invasive foreside, considering that signals that related to dedifferentiation usually originate from the tumor microenvironment. In these processes, Snail, as a member of the Snail family of transcriptional activators, modulates various other EMT phenotypes, such as the decreased expression of diverse epithelial markers including claudins, cytokeratin, and occludins. Furthermore, in the next step increase the expression of mesenchymal markers including vitronectin and fibronectin. Slug is another member of the Snail family of transcriptional activators and performs as a suppressing the epithelial phenotype in numerous cancer cells. Moreover, this transcriptional factor increases both migration and invasion of malignant gliomas [43,44]. These alterations cause MDR in GBM which is more considerable during chemotherapy (Figure 1).

## 3. MiRNAs

MiRNA plays a significant role in biological processes including cell proliferation and differentiation by targeting protein-coding mRNA at the posttranscriptional level [45]. MiRNA is one of the key regulators of the above-mentioned mechanisms of MDR by modulating target gene expression [46]. It is estimated that miRNAs control the translation status of more than 50% of the human genome. It means that a particular target gene can be controlled by multiple miRNAs, as well as a miRNA also can be involved in the regulation of various target mRNAs [47]. Multiple studies have highlighted the role of miRNAs in the process of carcinogenesis. However, intriguingly, it has appeared that the identical miRNA molecules might act as either suppressors and/or oncogenes, contingent on the organ or tissue [48]. The dysregulated miRNAs have been revealed to alter hallmarks of cancer including, evading growth suppressors, activating invasion, metastasis, angiogenesis, and resisting cell death.

## 4. Roles of MiRNAs in Glioblastoma

A broad range of microarray analyses has demonstrated that the expression profile of multiple miRNAs shows significant alteration in glioblastomas [49]. There is a long list of miRNAs, which facilitate cell growth, proliferation, invasion, metastasis, angiogenesis, evade immune destruction, and reprogram cellular energy in glioblastoma (Table 1). Exosomes (microvesicles) are extracellular vesicles (EVs) that contain various molecules such as DNA, mRNA, growth factors, oncogenic receptors, enzymes, and microRNA molecules. Tumor cells in aggressive glioblastomas can release these molecules, thereby inducing the oncogenic transformation of neighboring cells [50]. It is reported that the co-culture of U87MG astrocytoma cells and human neural stem cell-derived astrocytes led to the induction of malignant-like phenotypes in astrocytes acquired from tumor cells by inducing the expression of *GFAP*, *MMP-2*, *TGF-B1*, *SPARC*, and *CX43* [51]. Moreover, co-culturing of MSCs with U87MG simultaneously leads to a decrease in MMP inhibitor (*TIMP-2*) expression, indicating that U87MG could elevate a modification of the phenotype of neighboring astrocytes which may provide a significant change to the extracellular matrix of the tumor microenvironment and allow tumor invasion [52,53]. In addition, it was suggested that a certain miRNA was shared between normal and glioblastoma cells. Therefore, these studies proved that miRNAs play a direct role in glioblastoma malignancy.

## 5. The Most Frequent miRNAs Involved in the Signaling Pathway in Glioblastoma

It has been some miRNA being dysregulated in glioblastoma, including upregulation and downregulation which can bridge the widening gap between cancer treatment and failure. Some miRNAs play a crucial role in certain signaling pathways, with a particular mechanism that might be used as a sensitive and effective therapeutic approach for glioblastoma. Several important pathways have been identified as frequently genetically modified in cancer, including the EGFR/Ras/Raf/MEK/ERK pathways. Furthermore, this pathway plays a pivotal role in regulating cell proliferation and differentiation within the signaling network. The association of this pathway with some of the important microarrays in glioblastoma is considerable. For, instance, the *EGFR* gene expression has been altered in glioblastoma. *EGFR* amplification triggers downstream signaling pathways with moderate carcinogenicity. There are some new and important functional miRNAs in glioblastoma cells development:

### 5.1. MiR-218

miR-218 as a tumor-suppressive microRNA, which is decreased significantly in highly necrotic mesenchymal GBM. Studies revealed that reduced miR-218 levels confer GBM resistance to chemotherapy. Therefore, this miRNA was identified as a tumor suppressor gene in glioblastoma and regulated by RTK signaling in glioma cells [45,130]. RTK signaling pathway promotes tumor growth and plays an important role in tumor progression. Mathew et al. reported that in glioblastoma, *miR-218* inhibition increased multiple RTK activities through the regulatory feedback loop; this mechanism may suppress RTK signaling and ultimately result in the proliferation of glioblastoma. Briefly, the activation of the RTK signaling promotes the expression of *STAT3* which binds to the *miR-218* locus with B-cell lymphoma 2-associated transcription factor 1 (*BCLAF1*) and consequently inhibits the expression of *miR-218* and resulting in the suppression of glioblastoma proliferation [60]. In another study, glioblastoma samples with deep necrosis are substantially intensified in the mesenchymal transcriptional gene signature. In particular, hypoxic glioblastoma cells surrounding necrotic zones express high levels of C/EBP-β and C/EBP-δ, the mesenchymal transcription factors, indicating a link between hypoxia, necrosis, and specific mesenchymal transcription factors in glioblastoma cellular identity. The *miR-218* levels were found to be lower in highly necrotic and hypoxic glioblastomas than in less necrotic tissues. Furthermore, *miR-218* downregulation results in resistance to chemotherapy in glioblastoma. GSCs express more hypoxia-induced factors (HIFs), particularly, *HIF2*, which is required for GSC growth and survival. *MiR*-*218* is downregulated in mesenchymal glioblastomas that have high levels of necrosis and hypoxia and promotes either RTK or HIF activation. Therefore, the *miR*-*218*–RTK–HIF2α as an efficient signaling pathway influences mesenchymal glioblastoma that exhibits a high degree of aggressiveness. Furthermore, *HIF2α* knockdown diminishes *VEGF* expression, precludes GSC-induced angiogenesis, and therefore is considered a promising target for anti-GBM therapeutics. The upregulation of *miR-218* as a newly discovered tumor suppressor miRNA has proven to be a viable approach to dealing with chemoresistance [62].

### 5.2. MiR-7

*MiR-7* is one of the most potent tumor suppressors in GBM and has been shown to regulate proliferation, migration, and invasion. MiR-7 is expressed mostly in normal brain and pancreatic tissue, which illustrates a high degree of tissue specificity may be an ideal target for cancer therapy, particularly in GBM. Liu et al. reported that miR-7 can target multiple oncogenes including PI3K and Raf-1 by the EGFR pathway, bringing up a strong perception of the role of this miRNA in tumor cell proliferation. This miR is a common regulator of the important pathways including PI3K, ATK, Raf, MEK, and ERK. Bioinformatic studies revealed that there are four potential binding sites of miR-7 in the 3′-UTR of EGFR, PI3K, and Raf-1. This study confirmed that PI3K and Raf-1 mRNAs are direct targets of miR-7 through luciferase assay. However, there was no identified clear targeting relationship between EGFR and miR-7 in this experiment. As a result, miR-7 inhibits synchronously the PI3K/ATK and Raf/MEK/ ERK pathways via PI3K and Raf-1, which are placed downstream of EGFR. All of these findings imply that miR-7 can be a key factor and a potential therapeutic target in GBM [17,131,132].

### 5.3. MiR-21

**Figure 2:**
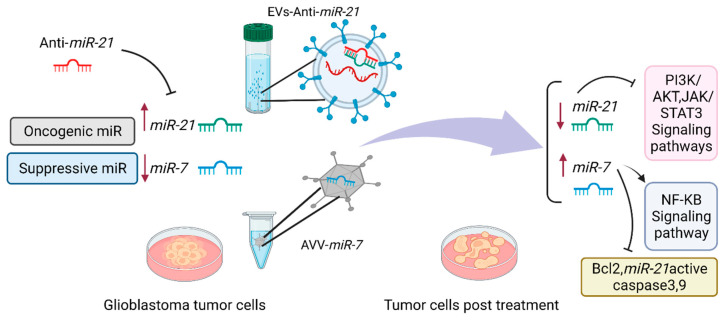
Treatment strategy based on *miR-21* and *miR-7* in glioblastoma. MiR-targeting therapy may involve activating or upregulating tumor suppressor miRs and inhibiting the function of oncomiRs. *MiR-21* is a key oncomiR, which is overexpressed in Glioblastoma. In contrast, *miR-7* is downregulated in Glioblastoma. Suppression of *miR-21* and upregulation of *miR-7* are crucial to targeting complementary pathways which inhibit glioblastoma growth and development. Assembling miRNA inhibitors or mimicking miRNAs to appropriate carriers including liposomes, extracellular vesicle (EVs), polymer-mediated delivery systems, viral vectors (VV) such as adenoviruses, and cell-based delivery systems and bacteriophage-based virus-like particles (VLPs) potentially inhibiting the function of the oncomiRs.

*MiR-21* has been consistently upregulated in glioblastomas and involved in a wide variety of biological pathways, promoting tumor cell survival and invasiveness [48]. This miR is the first one to be found in human glioblastoma. *STAT3*, as a part of the STAT family of transcription factors, has been illustrated to play a very essential role in glioma tumorigenesis via promoting angiogenesis, and invasion. Moreover, *miR-21* can be activated through a variety of other growth factor receptors and cytokines, including EGFR, IL-6R, JAK, and other kinases [133]. It has been suggested that reversion-inducing cysteine-rich protein with kazal motifs (*RECK*), a glycosylphosphatidylinositol-anchored membrane-bound regulator of matrix metalloproteinases (MMPs) as well as tissue inhibitor of metalloproteinase-3 (*TIMP3*), are both unlikely candidates to act as tumor suppressors during carcinogenic processes. In addition, these genes act as inhibitors of MMPs. It was discovered that *miR-21* upregulated in glioma samples with significantly higher stages, followed by a lower mRNA expression of *RECK* and *TIMP3* and subsequently, this process caused either reduction in *MMP-2* activity or cellular motility. MMPs disrupt the extracellular matrix and help glioma cells motility and metastasis [133]. MiR-21 enhances glioma cells resistance to carmustine (BCNU) and promotes cell cycle arrest in the G2/M phase, phosphatase and PTEN gene expression. Therefore, miR-21 inhibition increases the chemosensitivity of glioma cells [134]. Accordingly, *miR-21* inhibition enhanced the effectiveness of treatment. In recent studies, it has been demonstrated that inhibiting *miR-21* and inducing *miR-7* could be a promising strategy to inhibit parallel survival pathways and produce a synergistic effect in malignant glioma cells by inhibiting *BCL2,* PI3K/AKT, and Raf/MEK/ERK [135]. These findings provide evidence that miRNAs might be promising targets for glioblastoma treatment by regulating signaling pathways and opening the possibility for novel therapeutic approaches such as combined therapy to achieve synergistic inhibitions (Figure 2).

## 6. The Role of miRNAs in Glioblastoma MDR

### 6.1. MiRNAs That Target MDR Transporters

Tumor cells that overexpress drug transporters may be able to maintain low intracellular levels of cytotoxic chemotherapeutic agents by pumping them into extracellular space. MiRNAs play a significant role in regulating glioblastoma MDR by influencing the level of MDR transporter expression. *ABCG2* is a main member of ABC transporters with high expression levels in glioblastoma [136]. It has been demonstrated that *miR-328* targets and inhibits *ABCG2* in glioblastoma cells, thereby sensitizing the cells to chemotherapeutics [17]. It was also reported that upregulation of *miR*-*9* levels led to the inhibition of ABC transporters, including MDR1, *ABCC3,* and *ABCC6,* which reversed MDR in glioblastoma cells [137]. *MiR-381* is a common tumor suppressor miRNA that is downregulated in glioblastoma [138]. It has been demonstrated that *miR-381* overexpression effectively sensitized glioblastoma U251 cells to temozolomide by targeting various ABC transporters including *ABCG2*, *ABCC3*, and *ABCC5* [139]. *MiR-1268a* is another tumor suppressor miRNA with downregulated expression in glioblastoma. Li et al. [140]. reported downregulation of *miR-1268a* following temozolomide treatment in glioblastoma cells. The authors found that overexpression of *miR-1268a* suppressed protein translation of *ABCC1* and reversed upregulation of *ABCC1* due to temozolomide. Inversely, knockdown of *miR-1268a* increased *ABCC1* at the protein level and enhanced upregulation of ABCC1 with TMZ treatment [140].

### 6.2. MiRNAs Targeting Apoptosis

**Figure 3:**
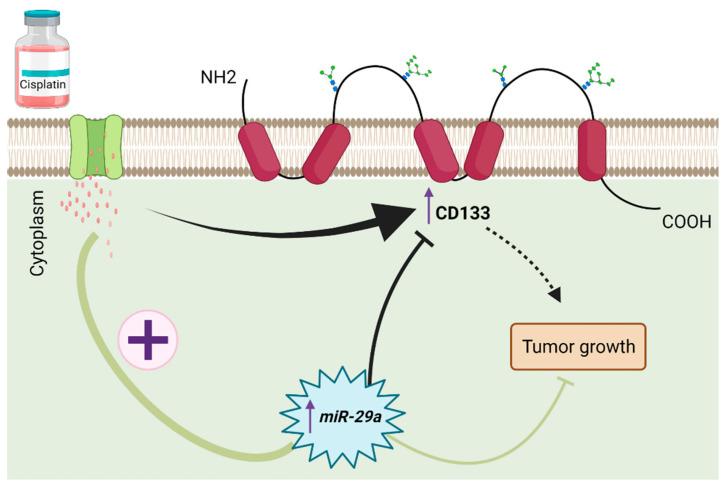
Treatment strategies for CD133+ cisplatin-resistant glioblastoma cells. CD133 as pentaspan transmembrane glycoprotein localized in protrusions of normal and cancer cells. Positive (CD133+) cells are regarded as tumor initiation cells in GBM. High CD133 expression confers resistance to glioma therapy and causes tumor growth. *MiR-29a* as a specific miRNA directly regulates CD133 expression. In fact, miR-29a promotes cisplatin-induced apoptosis via suppressing CD133 expression. Furthermore, using combination therapy including overexpression of miR-29a and cisplatin treatment substantially suppressed tumor growth in GBM than cisplatin treatment alone.

One of the most important mechanisms used by tumor cells in MDR is evading anti-cancer drug-induced apoptosis. Various microRNAs are ectopically expressed which disrupts apoptotic pathways and is implicated in the development of MDR in glioblastoma. For example, the inhibition of *miR-497* which is upregulated in glioblastoma cells, resulted in a significant increase in apoptosis and enhancement in the sensitivity of glioblastoma cells to temozolomide [141]. Treatment of glioblastoma cells with *miR-21* inhibitors leads to a significantly higher apoptotic rate than treatment with temozolomide alone, thus overcoming drug resistance [17]. As reported by Yang et al., upregulation of *miR-29a* in CD133+ glioblastoma cells increased cisplatin-induced apoptosis and decreased survival of CD133+ tumor-bearing mice after treatment with cisplatin [142] (Figure 3). Increasing the expression level of *miR-181b* led to a synergistic effect on temozolomide-induced apoptosis [143]. Further, miRNAs have been demonstrated to target the intrinsic and extrinsic pathways of apoptosis to reverse drug resistance in glioblastoma cells. Silencing an oncogenic microRNA, *miR-21*, in sunitinib-resistant glioblastoma cells led to an increase in the apoptotic rate of cancer cells by overexpressing *PTEN* and *PDCD4*, as well as increased activity of caspase 3/7, reversing MDR in these cells [144]. Shi et al. reported that combination treatment of glioblastoma cells with *miR-125b-2* and temozolomide potently increased cancer cells apoptosis through activation in the mitochondrial pathway by targeting *APAF-1*, CASPASE-3, BAX, BCL-2, and poly-ADP-ribose polymerase (PARP) [145]. *BCL-2* is a target of various microRNAs, such as *miR-181b-5p* and *miR-18*, that acts to overcome temozolomide-induced resistance in glioblastoma cells [146,147]. *MiR-155-5p*, *miR-221-3p*, *miR-21*, and *miR-125b* were reported to play critical roles in developing MDR in glioblastoma cells through targeting caspase-3 [148,149,150].

Mouse double minute 2 (MDM2) is an important negative regulator of the TP53 tumor suppressor which is also a direct target of miR-181b [151,152]. Sun et al. found that miR-181b overexpression sensitized U87 glioblastoma cell lines to temozolomide-mediated apoptosis by downregulating MDM2 [146]. Pro-apoptotic BCL-2 antagonist killer 1 (Bak1) is targeted by miR-125b, which increases the chemosensitivity of glioblastoma stem cells to temozolomide [153].

### 6.3. MiRNAs Targeting DNA Repair

Another well-established mechanism for miRNA-mediated MDR in glioblastoma involves targeting specific components of the DNA repair machinery.

The temozolomide is activated in the CNS by a chemical reaction which results in DNA methylation at various sites [154]. A DNA repair enzyme, O6-methyl-guanine-methyltransferase (*MGMT*), removes any methyl adducts from DNA [155]. It is important to note that *MGMT* has also been shown to contribute significantly to glioblastoma MDR [156]. *MiR-10a*, *miR-195*, and *miR-455-3p* are among the upregulated miRNAs in temozolomide-resistant glioblastoma cells [157]. *miR-181b* and *miR-181c* were also downregulated in patients with a glioblastoma that was resistant to temozolomide [158]. In both studies, it was established that the methylation status of *MGMT* was an independent predictor of response to temozolomide. Nie et al. [159] reported that *miR-198* was downregulated in glioblastoma patients. Patients with downregulation of this miRNA were more likely to have a poor prognosis. Moreover, in vitro and in vivo studies demonstrated that overexpression of *miR-198* was associated with enhanced chemosensitivity to temozolomide. This was accomplished by *miR-198* directly targeting *MGMT* and suppressing its protein translation. Therefore, *miR-198* induced chemosensitivity to temozolomide in glioblastoma by targeting *MGMT* [159]. In another study by Gao et al. [160], it was found that transfection of temozolomide-resistant glioblastoma cells with the *miR*-*370-3p*, which is downregulated in glioblastoma, enhanced the sensitivity of the cells to the anticancer drug by inhibiting self-repair capacity of tumor cells’ DNA. Based on their results *MGMT* is a direct target of *miR-370-3p* and that plays a critical role in the miRNA-mediated reversal of MDR in glioblastoma [160]. Non-homologous end-joining (NHEJ) is another DNA repair mechanism playing crucial functions in temozolomide sensitivity in glioblastoma.

An important component of this pathway is the XRCC4 protein, which has recently been discovered to be a direct target of *miR-151a* in resistant glioblastoma cells [161]. Zeng et al. [162] showed that low *miR-151a* levels in glioblastoma patients correlated with poor response to temozolomide therapy. Restoring *miR-151a* expression sensitized temozolomide-resistant glioblastoma cells through inhibition of XRCC4-mediated DNA repair [162].

### 6.4. MiRNA Regulating Cancer Stem Cells

MicroRNAs have been demonstrated to play a role in the CSCs-linked MDR in glioblastoma. *MiR-125b-2* is an oncogenic miRNA that is highly expressed in glioblastoma cells and GSCs. Shi et al. reported that treatment of GSCs with *miR-125b-2* inhibitors significantly increased stem cell sensitivity to temozolomide [8]. Furthermore, downregulation of *miR-21* inhibition in GSCs resulted in the suppression of cell proliferation and the induction of apoptosis, resulting in enhanced sensitivity to chemotherapeutic agents [163,164]. Cheng et al. found that *miR-132* plays a vital role in the development of resistance against temozolomide and induces the formation of CSC-like phenotypes in glioblastoma U87MG cells. Their finding indicated that *miR-132* inhibited the expression levels of tumor suppressor candidate 3 (*TUSC3*), which is downregulated in temozolomide-resistant U87MG cells (U87MG-res cells) and its renewal sensitized U87MG-res cells to temozolomide. This protein is able to inhibit the formation of GIC phenotypes in the U87MG-res cells. Hence, high expression levels of *TUSC3* were associated with the high sensitivity of cancer cells to temozolomide [165]. As discussed above, all of the studies have highlighted the important role played by miRNAs in the MDR induced by CSCs in glioblastoma.

## 7. MiRNA Targeting EMT

Glioblastoma MDR may be influenced by miRNAs targeting the key components of EMT [166]. *MiR-203* is a tumor suppressor miRNA, which is significantly downregulated in the resistant glioblastoma cells. This miRNA can bind to 3′-UTR *SNAI2*, as an embryonic protein with the ability to suppress E-cadherin transcription and induce EMT directly [117]. *SNAI2* was expressed at a higher level in glioblastoma-resistant cells. Transfection of *miR-203* in resistant cells inhibited *SNAI2* expression, reversing EMT and MDR in response to imatinib [117]. *MiR-26b* is also downregulated in temozolomide-resistant glioblastoma cells. It has been demonstrated that *miR-26b* can sensitize resistant cells to temozolomide by targeting *Wee-1* [167]. The expression of *Wee-1* plays an important role in regulating EMT and drug resistance by modulating the expression of sensible drug resistance genes and the activity of the MEK/ERK pathway [167]

## 8. Crosstalk between Signaling Pathways and miRNAs in Glioblastoma MDR

An accumulating number of recent studies have reported the mutual interaction between miRNAs and key components of various signaling pathways including epidermal growth factor, Wnt/β-catenin, nuclear factor kappa B (NF-κB), and PI3K signaling pathways in developing MDR in glioblastoma [168,169,170,171,172,173]. The EGFR is an important signaling pathway that ectopic activation of this receptor has been extensively characterized in glioblastoma cells [174]. Through this receptor, glioblastoma cells are induced to proliferate, differentiate, and survive [174]. Chen et al. [175] showed that *miR-181b* upregulation resulted in the significant enhancement in the chemo-sensitivity of glioblastoma cells to temozolomide through potentiating temozolomide-induced apoptosis. *MiR-181b* directly targets the EGFR, restoring EGFR decreased the suppressive effects of *miR-181b* and temozolomide treatment [175]. A separate study by Zhang et al. found that *miR-566* overexpression was associated with nimotuzumab resistance in glioblastoma cell lines. Moreover, treating cells with *miR-566* inhibitor decrease the EGFR pathway activity, reversing nimotuzumab resistance in glioblastoma cells [60]. Another study has demonstrated that exosomal *miR-1238* contributed to the development of temozolomide-resistance in glioblastoma cells in vitro and in vivo through significant activation of the EGFR-PI3K-Akt-mTOR pathways [176]. Zhang et al. [177] found that an increase in the expression levels of *miR-625* in glioblastoma cells inhibited cellular proliferation, induced apoptosis, and arrested cell cycle as well as suppressed tumor growth in the animal model of glioblastoma. Moreover, *miR-625* targeted and inhibited *AKT2*, thereby sensitizing cells to temozolomide [177]. The upregulation of *miR-423-5p* in glioblastoma cells was shown to lead to overexpression of signaling molecules such as p-AKT and p-ERK1/2. Therefore, this miRNA was responsible for glioblastoma MDR through activation of the AKT/ERK pathway [178]. NF-κB signaling is another signaling cross-talking with miRNAs and plays a key role in the glioblastoma MDR. Wang et al. showed that *miR-133a* induced TRAIL resistance in glioblastoma by inhibiting death receptor (DR)-5 expression and activating NF-κB signaling [84]. It was demonstrated that *miR-126-3p* and *miR-101* sensitized glioblastoma cells to temozolomide via targeting and inhibiting Wnt/β-catenin signaling [179,180].

## 9. Conclusions and Perspective

In cancer treatment, a major obstacle to patient treatment is the occurrence of MDR [9]. Various mechanisms contribute to the development of MDR during the treatment process [181]. It is noteworthy that miRNAs are important modulators of cellular pathways by regulating the expression of target genes during MDR; thus contributing significantly to the complexity of treatment failure in cancer progression, including glioblastoma [182]. The importance of some miRNAs for tumorigenesis and MDR mechanisms cannot be overstated. MiRNAs-mediated MDR in glioblastoma comes with a number of mechanisms including targeting MDR transporters, modulating apoptosis, targeting DNA repair machinery, controlling cancer stem cells, regulating EMT, and cross-talking with major oncogenic pathways. By targeting signaling pathways such as EGFR, RTK, and HIF2α, miRNAs have a direct effect on causing MDR and therefore increasing the effectiveness of glioblastoma treatment. There is no doubt that miRNAs are targeting the critical components of EMT, suppressing E-cadherin transcription and promoting EMT. Furthermore, miRNAs regulate signaling pathways such as epidermal growth factor, Wnt/β-catenin, NF-κB, and PI3K in the progression of MDR in glioblastoma. These studies demonstrate the importance of miRNAs in glioblastoma and glioma, but there are significant gaps in the knowledge in this area which need to be filled or improved. It will be necessary to clarify the role of potential miRNAs and their networking in modulating MDR mechanisms involved in glioblastoma. Furthermore, in the terms of therapeutics, there are not sufficient studies that address how miRNA applications can be translated for MDR glioblastoma patients to help move the bench-top research into clinical trials. There is a significant challenge in miRNA systemic delivery, especially which of natural and/or synthetic miRNA carriers work as the best systemic carrier for miRNA, their pharmacokinetics, as well as conducting related trials studies to understand their safety and effectiveness. In addition, in the terms of diagnosis and prognosis, profiling of miRNA needs more investigation to identify a miRNA signature for diagnosis and prognosis of resistance GBM via collecting the samples from body fluids such as serum, and CNS. This could be achieved by novel RNA-Based detection platforms such as Nonostring^®^.

Lastly but more importantly, we can suggest a potential application of miRNA studies in glioblastoma based on the analysis of the different studies discussed in this review. MiRNA can be used primarily for diagnostic and prognostic purposes in patients with glioblastoma. To recapitulate, by contemplating the subjects, miRNAs play an important role in the development of drug resistance in glioblastoma by modulating different types of important mechanisms and signaling pathways. A greater understanding of the roles of miRNAs in MDR glioblastoma will increase the number of miRNAs which may be nominated for replacement or blocking as potential therapeutic procedures. These candidates will be validated through extensive in vivo, in vitro, and ultimately clinical trials. In particular, miR-21 and miR-7 are momentous and functional miRNAs in MDR glioblastoma cells, which should be excellent candidates for further investigation both in vivo and in vitro. MiR-21 is one of the most prominent oncogenic miRNAs in cancer, especially glioblastoma. Blocking this miRNA with anti-miR or miRspong is a potential strategy to decrease its oncogenic function. Moreover, inhibiting miR-21a is an effective therapeutic approach, especially for GCS (CD133+) cells, which are the main population for GBM relapse. In contrast, MiR-7 is a tissue-specific miRNA that is significantly reduced in GBM cells, and its replacement may prove to be an ideal treatment strategy for the disease. Furthermore, combining commonly used GBM chemotherapeutic agents or small inhibitor molecules with miRNA-based therapeutics may have the potential to overcome patient resistance. The combination approach requires extensive knowledge of the functional properties of therapeutic agents and miRNAs; selecting miRNAs that complement agent function is crucial in this approach.

## Figures and Tables

**Table 1:** Involvement of miRNAs in glioblastoma.

miRNA	Expression in Glioblastoma	Targets Genes	Effects	Ref.
Facilitate cell growth and proliferation in glioblastoma				
Sustaining Proliferative Signaling				
*miR-7*	Down-regulated	*EGFR*	transfection with *miR-7* decreased viability and invasiveness	[54]
		*AKT*		
*miR-128*	Down-regulated	*EGFR*	correlates with aggressive human glioma subtypes	[45]
		*PDGR*	*miR*-*128* inhibits growth and mediates differentiation	
*miR-133*	Down-regulated	*EGFR*	decreased cell growth and increased cell apoptosis	[55]
*miR-218*	Down-regulated	*RTK*	decreased tumor burden and reduced survival	[45]
		*HIF2α*		
*miR-219-5p*	Down-regulated	*MAPK*	inhibit the proliferation, anchorage-independent growth and migration	[56]
		*PI3K*		
		*EGFR*		
*miR-491-5p and -3p*	Down-regulated	*EGFR*	decrease cellular proliferation and invasion	[57]
		*CDK6*	inhibit the propagation of glioma stem cells	
		*BCL-XL*		
		*IGFBP2*		
		*CDK6*		
*miR-7*	Down-regulated	*PI3K*	Inhibit cell cycle and cell growth	[58]
		*AKT*		
		*RAF-1*		
		*MEK 1/2*		
		*cyclin D1*		
		*EGFR*		
*miR-34a*	Down-regulated	*SMAD4*	Decrease cellular proliferation and invasion	[59]
		*PDGFRA*	*miR-34a* expression level is shown to be prognostic	
*miR-218*	Down-regulated	*EGFR*	Decrease cellular proliferation	[60]
		*PLCγ1*		
		*PIK3CA*		
		*ARAF*		
		*PDGFRα*		
		*RSK2*		
		*S6K1*		
		*STAT3*		
		*BCLAF1*		
*miR-410, miR-144-3p, and miR-34a*	Down-regulated	*C-MET*	The overexpression of these miRs produces anti-proliferative effects	[58,61]
*MiR-126, let-7a, and miR-622*	Up-regulated	*KRAS*	restrain glioma cells’ proliferation	[62]
*MiR-124*	Up-regulated	*R-RAS*	governs glioma growth and angiogenesis and enhances chemosensitivity	[63]
		*N-RAS*		
*MiR-143*	Down-regulated	*N-RAS*	acts as a tumor suppressor	[62]
			enhances temozolomide-induced apoptosis in glioma	
*let-7e*	Down-regulated	*N-RAS*	Repress tumor function by decreasing proliferation, migration and invasion while promoting apoptosis	[64]
*miR-17-5p, miR-19a/b, miR-21, miR-1908, miR-494-3p, miR-10a/10b, miR-23a, and miR-26a*	Down-regulated	*PTEN*	Inhibit tumor growth	[65,66]
*miR-542-3p*	Down-regulated	*AKT*	Suppress tumor cell proliferation and invasion	[67]
*MiR-199a-3p*	Down-regulated	*mTORC1 and mTORC2*	decrease glioma cell proliferation	[68]
*MiR-34a*	Down-regulated	*RICTOR*	Inhibit cell proliferation and tumor growth of glioma stem cells	[69]
		*AKT*		
		*WNT* signaling		
Evading Growth Suppressors				
*miR-10b*	Up-regulated	*TP53*	promotes growth, invasiveness, and angiogenesis and inhibits apoptosis	[70]
		*MMP14*		
		*UPAR*		
		*RHOC*		
		*HOXD10*		
*miR-25 and -32*	Down-regulated	*TP53*	inhibited growth of the glioblastoma multiforme cells	[71]
		*MTOR*		
		*MDM2*		
		*TSC1*		
*miR-17*	Up-regulated	*PTEN*	promoted cell motility, invasion, and tube-like structure formation	[72]
		*HIF1*		
		*VEGF*		
*MiR-217*	Up-regulated	*YWHAG*	enhances the proliferation of cells	[73]
		*MDM4*		
		*TP53*		
*MiR-26a*	Up-regulated	*RB*	promotes GBM formation	[71]
*MiR-329 and miR-320*	Down-regulated	*E2F1*	inhibit cell proliferation	[74]
*MiR-195*	Down-regulated	*CCND1*	block GBM cell proliferation by inducing G1-S arrest	[75]
		*CCNE1*		
*let-7b, miR-15b, miR-34a, and miR-340*	Down-regulated	*CCND1*	block cell cycle and proliferation	[76,77,78]
*miR-34a, miR-107, miR-138, miR129-3p, miR-29b-1, miR-218, miR-129-1, miR-340, miR-491-3p/5p*	Down-regulated	*CDK6*	inhibit cell cycle of GBM cells	[45,76,79,80,81]
*MiR-10b*	Down-regulated	*CDKN2A/p16INK4A*	arrest the cell cycle	[82]
*miR-138*	Down-regulated	*CDK6*	inhibit cell proliferation	[82]
		*EZH2*		
		*PRB-E2F1*		
Resisting Cell Death				
*miR-21*	Up-regulated	*FASL*	suppressed the apoptosis in GBM stem cells (GSCs)	[83]
*miR-133a*	Up-regulated	*DR5*	suppressed the cells’ apoptosis	[84]
*miR-363 and miR-582-5p*	Up-regulated	*BIM*	inhibit GSC apoptosis to promote GSC growth	[85]
		*CASPASE-3*		
		*CASPASE-9*		
*miR-21 and miR-30b/c*	Up-regulated	*CASPASE-3*	prominently inhibited TRAIL-induced apoptosis	[86]
		*TAP63*		
*miR-148a, miR-363, miR-92a*	Up-regulated	*BIM*	decrease apoptosis	[85]
*MiR-16, miR-34a, and miR-429*	Down-regulated	*BCL-2*	increase apoptosis to suppress proliferation in glioma cells	[87,88,89]
*MiR-29b*	Down-regulated	*BCL2L2*	induce apoptosis in GBM cells	[90]
*MiR-139*	Down-regulated	*MCL-1*	promoted apoptosis related to TMZ	[91]
*miR-153*	Down-regulated	*MCL-1, BCL-2, and IRS-2*	inhibited survival and promoted apoptosis	[92]
Enabling Replicative Immortality				
*miR-141*	Down-regulated	*JAGGED1*	suppressed the self-renewal of GSCs	[93]
*miR-181a*	Down-regulated	*NOTCH2*	suppressed GSCs formation and proliferation and increased apoptosis of GSCs	[94]
*miR-182*	Down-regulated	*HIF2α*	reduced the aggressive phenotype of GSCs	[95]
*miR-148a and miR-31*	Up-regulated	*HIF1α*	activated Notch signaling to maintain potential of GSCs	[96]
		*HIF1AN*		
*miR-128*	Down-regulated	*PDGFRα*	significantly suppressed proliferation in GSCs	[97]
		*EGFR*		
*miR-608*	Down-regulated	*MIF*	suppressed proliferation, invasion and promoted apoptosis in GSCs	[98]
*MiR-152*	Down-regulated	*KLF4*	exerts tumor-suppressive effects	[99]
*miR-101 miR-608*	Down-regulated	*KLF6*	exerts tumor-suppressive effects	[100]
*MiR-449a*	Down-regulated	*MAZ*	blocks proliferation and induces apoptosis in GSCs	[101]
*miR-29a*	Down-regulated	*QKI-6*	repressed the malignant behavior of GSCs	[102]
		*WTAP*		
*miR-663*	Down-regulated	*CXCR4*	effectively suppressed the invasion and proliferation of GBM cells	[103]
*miR-137*	Down-regulated	*RTVP-1*	inhibited the self-renewal of GSCs	[104]
Activating Invasion and Metastasis				
*miR-663, miRNA-181c, and miR-564*	Down-regulated	*TGF-β1*	suppress the invasion.	[105,106,107]
*MiR-373 and miR-520c*	Down-regulated	*TGFBR2*	suppress the invasion of GBM	[108,109]
*miR-211 and miR-491-5p*	Down-regulated	*MMP-9*	suppress the invasion of GBM	[58]
*MiR-152*	Down-regulated	*MMP-3*	suppresses the invasion of GBM	[110]
*MiR-16*	Down-regulated	*NF-κB1*	inhibits the invasion of GBM cells.	[89]
*MiR-203*	Down-regulated	*ROBO1*	suppresses the migration of glioma cells	[111]
		*ERK*		
		*MMP-9*		
*miR-218*	Down-regulated	*MMP-9*	inhibit the invasion of GBM	[112]
		*LEF1*		
*MiR-7*	Down-regulated	*FAK*	suppresses the invasion of GBM	[112]
*MiR-21*	Up-regulated	*TIMP-3* and *RECK*	enhances the expression of MMPs and glioma cell invasion	[113]
*miR-20a and miR-106a*	Up-regulated	*TIMP-2*	increase the invasion of GBM	[114]
*MiR-124*	Down-regulated	*CAPN4*	inhibited the invasion of glioma cells.	[115]
*Let-7g-5p*	Down-regulated	*VSIG4*	inhibited GBM cell invasion and migration	[116]
*MiR-203*	Down-regulated	*SNAI2*	inhibits the invasive and migratory capacity of GBM cells	[117]
*MiR-590-3p*	Down-regulated	*ZEB1* and *ZEB2*	suppresses the invasive and migratory capacity of GBM cells	[118]
Inducing Angiogenesis				
*MiR-23b, miR-566*	Down-regulated	*VEGF-A*	decrease angiogenesis capacity in tumor	[112,119]
		*VHL*		
*MiR-128*	Down-regulated	*P70S6K1*	suppress tumor angiogenesis	[120]
*MiR-125b*	Down-regulated	*MAZ*	inhibited VEGF-mediated angiogenesis	[121]
Evading Immune Destruction				
*miR-124*	Down-regulated	*STAT3*	reversed immune suppression associated with T cells	[122]
			Exhaustion of T cells (CD4+ and CD8+) completely counteracted the role of *miR-124* in the inhibiting glioma	
*miR-146b-5p*	Down-regulated	*TRAF6*	Regulate immunosuppression and macrophage polarization	[123]
*miR-31*		*TRADD*		
*miR-17-92*	Down-regulated	*TGFBR2*	activated CD8+ CD44+ memory T cells (Th1 CD8+ T cells) which then secreted IFN-γ and increased resistance of CD8+ T cells to the immunosuppressive effects of *TGF-β1*	[124]
*miR-138*	Down-regulated	*CTLA-4*	reversed the immunosuppressive effects to exert antitumor properties	[125]
		*PD-1*		
*miR-20a, miR-93, and miR-106b*	Down-regulated	*NKG2DL*	improved NK cell-mediated cytotoxicity	[126]
Reprogramming Cellular Energetics				
*miR-106a*	Down-regulated	*GLUT3*	blocked glucose uptake in GBM cells	[127]
*miR-143*	Down-regulated	*HK2*	suppressed glycolysis	[112]
*miR-326*	Down-regulated	*PKM2*	suppressed glycolysis	[128]
*let-7a*		*C-MYC*	inhibit glucose metabolism and the growth of gliomas	[129]
		*HNRNPA1*		
		*PKM2*		
